# Everybody's Page

**Published:** 1891-06-06

**Authors:** 


### EVERYBODY'S PAGE.

De Schweinitz's recent experiments show that when quinine is given hypodermically to dogs, in quantities varying from one to four grains to the pound, blindness, usually accompanied by other general disturbance, is apparent from three to fourteen hours. Each dog experimented upon died from the effect of the drug.

The School Board evening classes seem to be gaining in popularity, and to be meeting with no small success. The fee for the week is not ruinous, amounting, as it does, to the sum of 1*d.* Though the classes meet on one evening in a week only, the course of instruction, which embraces physical exercises is one which cannot fail to benefit the more weakly children.

The *Annales d'Hygiene* is not altogether complimentary to the self-restraint of public men in America, where banqueting in public life is alleged to be an enemy to old age. Prominent Americans, according to this journal, have usually been abstemious and hard-working till, as the direct results of this very abstemiousness and hard work, they have become prominent. Then they give rein to the appetite until their career is summarily cut short by gout or degeneration of one or more internal organs. No doubt a man of sixty and upwards who does not materially modify his life of forty so as to live quietly and avoid excitement is likely at any moment to pay prematurely his debt to nature.

Those who were beguiled by the three hot days at the beginning of the month to discard their warm clothing will have been bewailing in uncomfortable leisure the temerity which led them to flout the adage, "Cast not a clout till May is out." Never has a saw been more worthy of respect than this. What subtle irony there is in the application of the epithet "merrie" to the month of May in England!

The abolition of compulsory taxation, and the institution in its place of a voluntary system, sounds pleasant, but who would be so weak as voluntarily to tax himself? The gentlemen who wish to abolish compulsion were not very clear as to the method by which they intend to accomplish the proposed reform, and as what they had to say was said after *breakfast*, and not after dinner, there is not any good or explainable excuse for this haziness of ideas.

An excellent plan has been adopted by the municipal authorities of Rome with a view to preventing adulteration, which other municipalities might follow with advantage. Recognising the fact that the public can glean but little knowledge from the annual reports of food inspectors, they have required the names of all makers and sellers of alimentary substances injurious to health, or adulterated, to be published in the daily papers.

